# Supplementary figures and images for: LACTB mRNA expression is increased in pancreatic adenocarcinoma and high expression indicates a poor prognosis
Source: PLoS One. 2021 Jan 28;16(1):e0245908. doi: 10.1371/journal.pone.0245908 (PMC7842907; doi:10.1371/journal.pone.0245908)

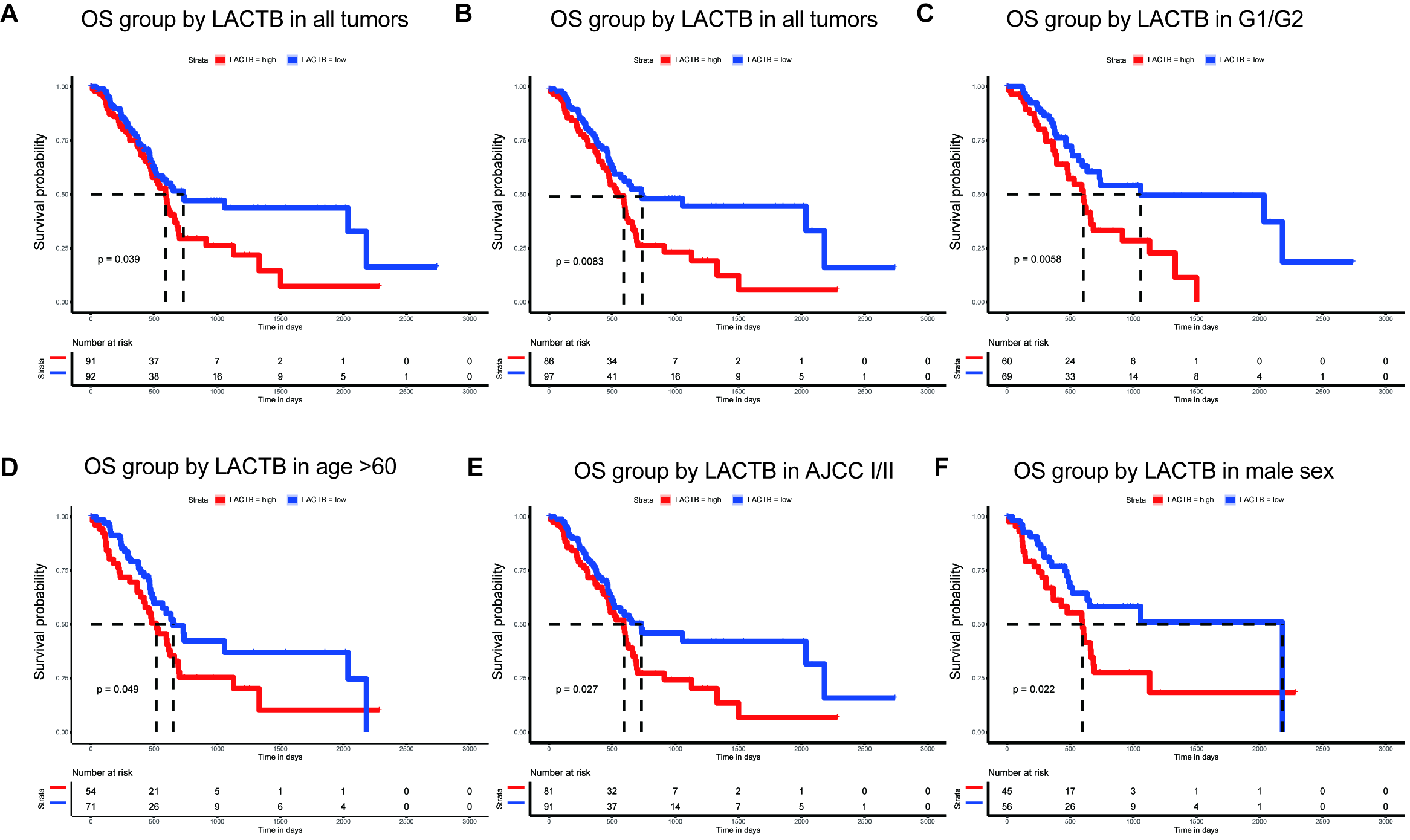

Supplement: S1 Fig — OS values were analyzed in relation to the mRNA expression level of LACTB in all tumors and subgroups of PAAD patients. OS analyses of (A) all tumors (divided according to the median LACTB expression levels), (B) all tumors (divided according to the best separation), (C) G1 +G2 stage, (D) age ≥ 60 years, (E) AJCC stage I/II and (F) male sex. (TIF) [file pone.0245908.s001.tif]

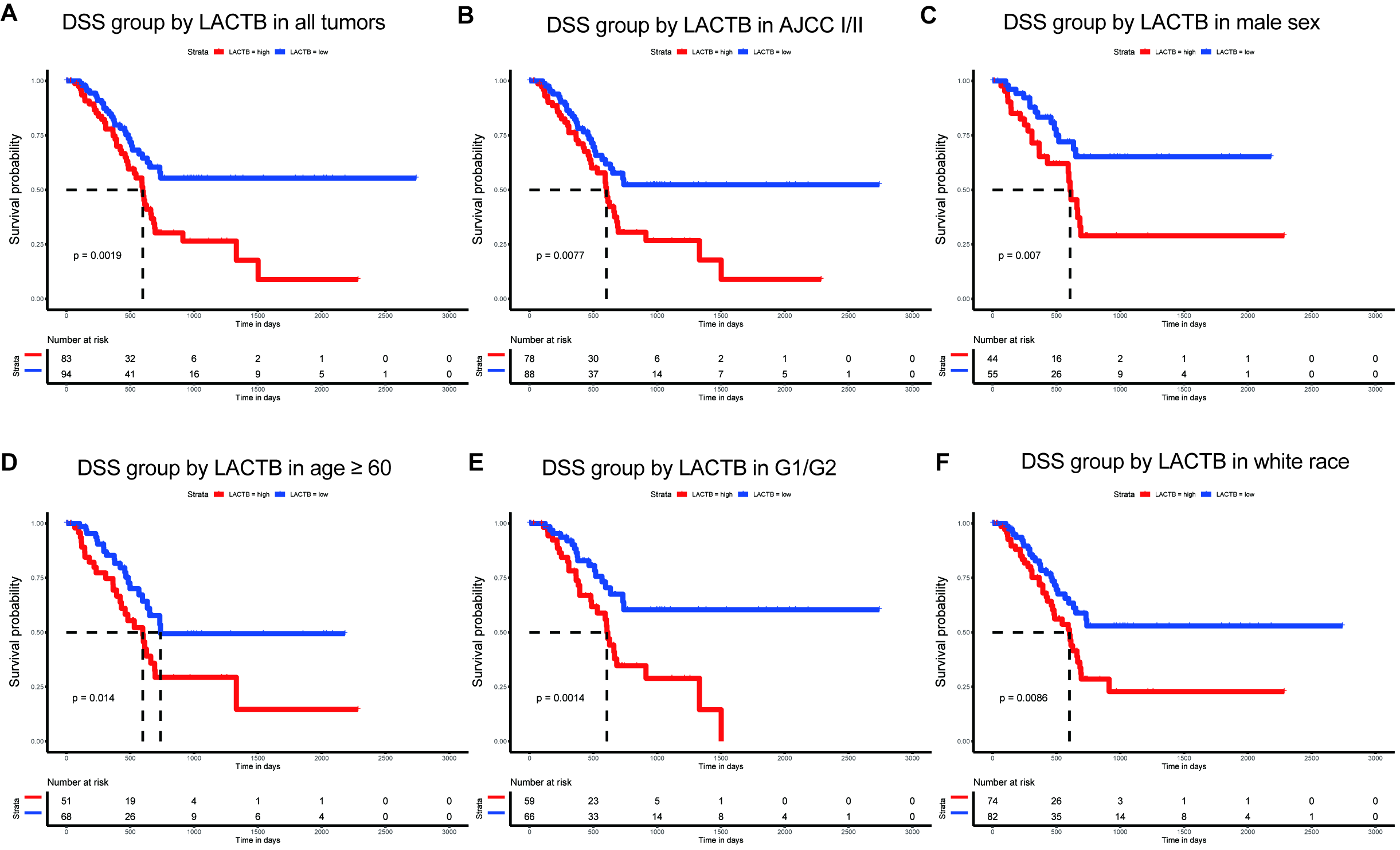

Supplement: S2 Fig — DSS values were analyzed in relation to the mRNA expression level of LACTB in all tumors and subgroups of PAAD patients. DSS analyses of (A) all tumors, (B) AJCC stage I/II, (C) male sex, (D) age ≥ 60 years, (E) G1 +G2 stage and (F) white race. (TIF) [file pone.0245908.s002.tif]

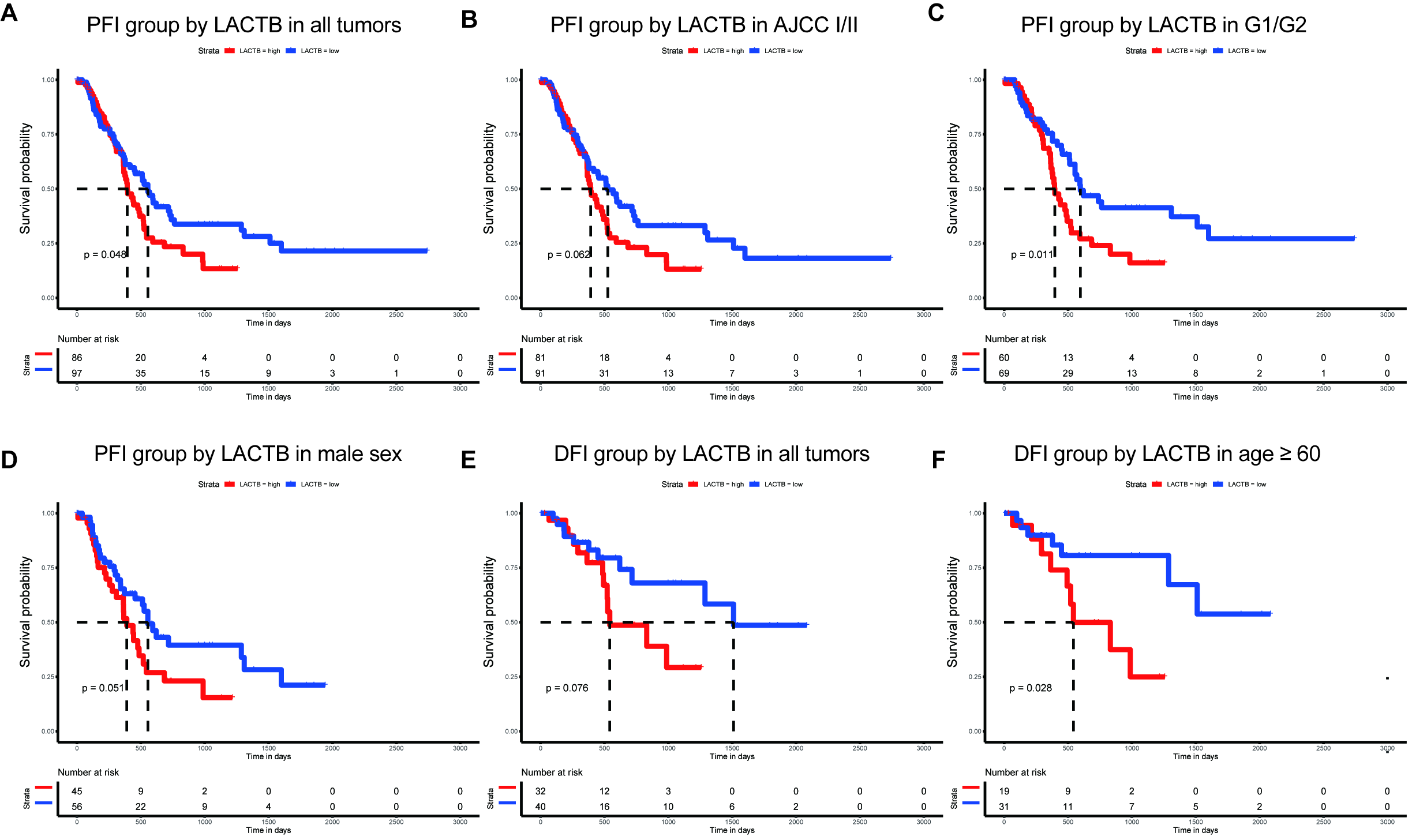

Supplement: S3 Fig — PFI and DFI values were analyzed in relation to the mRNA expression level of LACTB in all tumors and subgroups of PAAD patients. PFI analysis of (A) all tumors, (B) AJCC stage I/II, (C) G1 +G2 stage, and (D) male sex; DFI analysis of (E) all tumors and (F) age > 60 years. (TIF) [file pone.0245908.s003.tif]

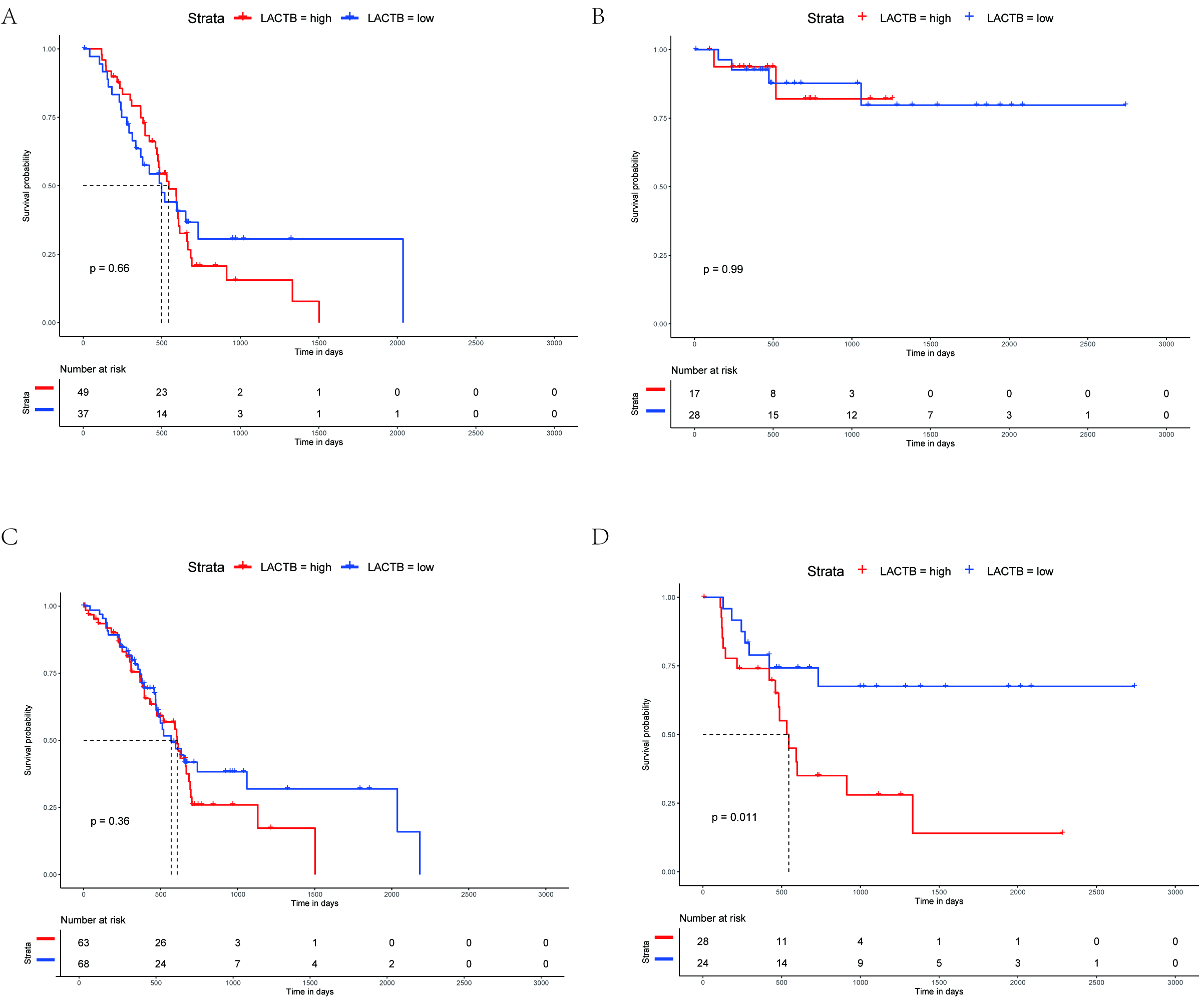

Supplement: S4 Fig — OS analyses of (A) positive margins of resection (B) negative margins of resection, (C) tumor location with head of pancreas (D) tumor location without head of pancreas. (TIF) [file pone.0245908.s004.tif]
