# Supplementary material for: LACTB mRNA expression is increased in pancreatic adenocarcinoma and high expression indicates a poor prognosis
Source: PLoS One. 2021 Jan 28;16(1):e0245908. doi: 10.1371/journal.pone.0245908 (PMC7842907; doi:10.1371/journal.pone.0245908)
Supplement: S1 Table — (DOCX) [file pone.0245908.s005.docx]

|  | **High (N=36)** | **Low (N=55)** | **P-value** |
| --- | --- | --- | --- |
| **Age** |  |  |  |
| age <60 | 20 (55.6%) | 26 (47.3%) | 0.577 |
| age >= 60 | 16 (44.4%) | 29 (52.7%) |  |
| **Gender** |  |  |  |
| female | 19 (52.8%) | 17 (30.9%) | 0.0619 |
| male | 17 (47.2%) | 38 (69.1%) |  |
| **His_Grade** |  |  |  |
| II | 34 (94.4%) | 45 (81.8%) | 0.154 |
| III | 2 (5.6%) | 10 (18.2%) |  |
| Tumor stage |  |  |  |
| T2 | 29 (80.6%) | 42 (76.4%) | 0.362 |
| T3 | 7 (19.4%) | 10 (18.2%) |  |
| T1 | 0 (0%) | 3 (5.5%) |  |
| Lymph node status |  |  |  |
| N0 | 20 (55.6%) | 30 (54.5%) | 1 |
| N1 | 16 (44.4%) | 25 (45.5%) |  |
| Metastasis status |  |  |  |
| M0 | 36 (100%) | 53 (96.4%) | 0.67 |
| M1 | 0 (0%) | 2 (3.6%) |  |
| **AJCC** |  |  |  |
| IB | 15 (41.7%) | 20 (36.4%) | 0.568 |
| IIA | 5 (13.9%) | 6 (10.9%) |  |
| IIB | 16 (44.4%) | 25 (45.5%) |  |
| IA | 0 (0%) | 2 (3.6%) |  |
| IV | 0 (0%) | 2 (3.6%) |  |
| **Tumor_site** |  |  |  |
| Body_or_Tail | 10 (27.8%) | 17 (30.9%) | 0.0405 |
| Head | 26 (72.2%) | 30 (54.5%) |  |
| Other | 0 (0%) | 8 (14.5%) |  |
| **status** |  |  |  |
| 0 | 24 (66.7%) | 44 (80.0%) | 0.236 |
| 1 | 12 (33.3%) | 11 (20.0%) |  |
| **months** |  |  |  |
| Mean (SD) | 28.3 (24.1) | 26.0 (29.6) | 0.689 |
| Median [Min, Max] | 21.5 [0.600, 80.0] | 11.0 [0, 87.0] |  |

Abbreviations: AJCC = american joint committee on cancer
