# Supplementary material for: LACTB mRNA expression is increased in pancreatic adenocarcinoma and high expression indicates a poor prognosis
Source: PLoS One. 2021 Jan 28;16(1):e0245908. doi: 10.1371/journal.pone.0245908 (PMC7842907; doi:10.1371/journal.pone.0245908)
Supplement: Attachment — Submitted filename: Response to Reviewers.docx [file pone.0245908.s009.docx]

**Journal Requirements:**

**1. Please ensure that your manuscript meets PLOS ONE's style requirements, including those for file naming. The PLOS ONE style templates can be found at**

The article format has been modified as required

**2. Please provide the accession numbers and/or URLs of the datasets obtained from TCGA and GTEx.**

All URLs a were supplied on data availability.

**3. We note that you obtained a tissue microarray from Shanghai Outdo Biotech Co. Please ensure it is clear to readers that your study did not involve prospective collection of tissue samples. Specifically, please ensure that you do not refer to "patients recruitment".**

We have modified the statement accordingly to ensure that the reader understands correctly, we have changed “patients recruitment” to “samples collected”.

**4. In your ethics statement in the manuscript and in the online submission form, please provide additional information about the patient data used in your study. Specifically, please ensure that you have discussed whether all data were fully anonymized before you accessed them.**

All additional information on the use of patients has been submitted as additional information and full anonymity is ensured.

**5. To comply with PLOS ONE submission guidelines, in your Methods section, please provide additional information regarding your statistical analyses. For more information on PLOS ONE's expectations for statistical reporting, please see** [**https://journals.plos.org/plosone/s/submission-guidelines.#loc-statistical-reporting**](https://journals.plos.org/plosone/s/submission-guidelines.#loc-statistical-reporting)**.**

To facilitate reproduction of article results, all analysis data have been provided as additional information.

**Reviewer #1:**

**Minors :**

(1) **Title is ambiguous, please specify if high or low levels are associated to poor prognosis.**

To eliminate ambiguous, we changed the title to “LACTB mRNA expression is increased in pancreatic adenocarcinoma and high expression indicates a poor prognosis”

(2) **Include an updated information about 5-years survival rates, mortality, presence of metastasis, accurate prognosis biomarkers, etc. Please try to provide an exact number in each case and avoid generalities.**

All the above-mentioned issues have been corrected

(3) **In Table 1 headline please refer to TCGA cohort. In addition, provide an accurate stratification of patient by ethnicity, white and non-white is a rough estimation. Please include margin positiveness in case of resected patients and include this variable in analyses. And include in abbreviations the meanings of NA, G, T, N and M.**

**All the above-mentioned issues have been corrected, eg: The stratification of patient by ethnicity has been updated. T = Tumor stage, N = Lymph node status, M = Metastasis status, G = Histologic grade, NA = Not available.**

(4) **Explain the cut-off point when mRNA expression levels are used.**

The samples were divided into high-risk and low-risk groups based on the median LACTB expression levels.

(5) **In "High LACTB mRNA is associated with a poor survival rate" section OS, DSS, DFI, and PFI analyses was assessed in a subgroup of PAAD patients. Please explain inclusion criteria of this subgroup.**

To ensure more accurate results, we expected to include as many patients as possible in the analysis; however, not all patients in the TCGA database had OS, DSS, DFI, and PFI information collected, so we only analyzed patients for whom OS (183), DSS (177 patients), DFI (72 patients), and PFI (183 patients) information existed.

(6) **Please check the order of the variables included in the uni- and multi-variate analyses e.g. HR of high vs Low expression LACTB is 1.72 and HR of stage IV/III vs I/II is 0.67 what does not make any sense.**

We repeated the calculations, but came to the same conclusion, suggesting that the results might be due to the small sample size of AJCC stage III/IV patients (eight patients); thus, expanding the sample size might provide a more valid result.

(7) **Figure legend of Fig.2 is not well described**

Fig 2. Univariate and multivariate regression analyses of the relation between the expression of LACTB and clinicopathological characteristics regarding OS (A) and DSS (B) in the TCGA cohort. We used the COX regression algorithm and p-values less than 0.05 in the univariate analysis were included in the multifactor analysis

**Majors**

1. **The study has been carried out with a high heterogeneous TCGA cohort that includes resectable and non-resectable tumors and several tumor locations. Please re-analyse with selected patients or do a** **stratification according to stage,** **tumor location and** **positive margins of resection.**

The stratification according to stage, tumor location and positive margins of resection were provided in supplement figures.

2. **Include clinico-pathological characteristics of 98 primary tumors included for protein validation.**

The data was provided in supplement Table 1.

3. **Include a uni- and multi-variate analysis for survival of the primary tumors included for protein validation.**

We found no predictive value for LACTB at the protein level. The figure was provided in supplement Table 2 and supplement Table 3. All information about the data was supplied in supplement Table 4.

4. **Since LACTB presents a clear cytoplasmic staining, score has been performed with both intensity of expression and % of positive staining cells. Please justify why just intensity has been taken into consideration.**

To facilitate subsequent statistical analysis, LACTB was scored according to staining intensity from 1+ to 3+. A score of 1+ to 2+ was defined as low LACTB expression, whereas a score of 3+ was defined as high LACTB expression.

5. **Association/correlation with leading genes intersecting with LACTB must be validated at least at protein expression.**

CDC20, CDK4, MCM6, MAD2L1, MCM2 and MCM5 were leading genes that intersected in signal transduction pathways by gene set enrichment analysis (GSEA). There is a strong correlation between these genes at the molecular level and LACTB. A PPI network was constructed using the genemania online tool(https://genemania.org/) and showed that these target genes and LACTB exhibited complex interactivity with each other (Fig 5). Therefore, we predict that LACTB may function biologically together with these genes. However, more experiments are needed to validate the protein levels.

**Reviewer #2:**

1. **Figures were not visible for review and that hindered the ability to review any Fig from the paper. Please paste the figures within the submitted draft so that it can be reviewed**

All figures have been provided.

2. **Under the section ImmunoHistochemical Staining it says: "For IHC analysis, a tissue microarray including 98 primary pancreatic cancer tissues and 68 adjacent noncancerous pancreatic tissues...". It is not clear from the statement whether the cancerous and non cancerous samples were taken from the same patient/different patient? Are the two sample datasets mutually exclusive vis-a-vis PAAD? If they were from the same set of individuals, is there a reason for only 68 noncancerous tissue samples versus 98 cancerous.**

We apologize for using such a puzzling description. We have made the following corrections: For IHC analysis, a tissue microarray including 98 primary pancreatic cancer tissues and 68 noncancerous pancreatic tissues was obtained from Shanghai Outdo Biotech Co., Ltd. (Shanghai, People’s Republic of China; Category no: HPan-Ade170Sur-01).

3. **Also how is the PAAD patient table broken down by age/ethnicity etc..**
   To facilitate statistical analysis, we set the age to 60 as the cutoff point and White and non-white as ethnicity.
4. **Table 2 provides a breakdown of the LACTB expression but the % values will need to be explained. I could not figure out how the numbers 62.8% and 73.2 were calculated.**

That's for the chi-square test, represents in high LACTB expression patients 62.8% patients more than 60 years old and 37.2% patients less than 60 years old; In addition, in low LACTB expression patients, 73.2% patients more than 60 years old 26.8% patients less than 60 years old.

5. **Here: "The chi-square test was performed to examine the relationship between LACTB mRNA expression and clinical data"
   This section, what "clinical data" are we comparing to? there is no reference. The Chi Square test will need to be clarified better if the samples aren't assumed to be mutually exclusive..**

To prevent misunderstandings, we have modified the statement as follows: The chi-square test was performed to examine the clinical relationship between high and low LACTB mRNA expression patients
