# Supplementary material for: LACTB mRNA expression is increased in pancreatic adenocarcinoma and high expression indicates a poor prognosis
Source: PLoS One. 2021 Jan 28;16(1):e0245908. doi: 10.1371/journal.pone.0245908 (PMC7842907; doi:10.1371/journal.pone.0245908)
Supplement: Attachment — Submitted filename: Response to Reviewers.docx [file pone.0245908.s010.docx]

**We thank the reviewers for their valuable comments, all of questions have been carefully reviewed and revised.**

**Reviewer #1:**

**1.-Please describe in "Results" section those findings after survival analyses according to tumor location and positive margins of resection and discuss them in "Discussion" section.**

We have added the section as follows:

**Results**: OS analysis found that patients with positive margins of resection (P = 0.66), patients with negative margins of resection (P = 0.99) and patients with head of pancreas (P = 0.36) showed no significant difference between two LACTB mRNA expression groups (S4 Fig.1-3). However, patients without head of pancreas showed a better OS with high LACTB mRNA expression (P=0.011) (S4 Fig.4).

**Discussion**: However, patients without head of pancreas showed a better OS with high LACTB mRNA expression. Therefore, our analysis of the physiological function of LACTB may also differ in different subgroups and need further analysis. This discrepancy suggests that the real roles of LACTB vary in different cancer types and that other unreported mechanisms may be involved in the effects of LACTB in PAAD.

**2.-Please include a survival analysis of R0 patients stratified by LACTB expression (mRNA and protein), and in R1 patients stratified by LACTB expression (mRNA and protein).**

We interpret R0 as patients with negative margins of resection, and R1 as patients with positive margins of resection. However, the protein data of LACTB expression were obtained from Shanghai Outdo Biotech Co., Ltd. (Shanghai, People’s Republic of China; Category no: HPan-Ade170Sur-01), they did not record the corresponding information about margins of tumor resection. Therefore, we present the results of OS analyses of positive margins of resection and negative margins of resection between high and low LACTB mRNA expression in supplement figure 4. And added the corresponding text description in the **Results and Discussion** section.

**3.-Previous major point 5 has not been amended. As least, include in the "Results" section that more experiments are needed to validate the protein levels**

We regret that we were unable to complete the validation at the protein level and therefore we note in the results section as follows: more experiments are needed to validate the protein levels in the future.

**4.-Include in Table S2 whether patients are R0 or R1
5.-Include in Table S4 the R0 or R1 status of each patient.**

Here we make the following explanation: Table S2 and Table S4 showed the clinico-pathological characteristics and predictive value of LACTB in protein level in PAAD. However, the protein data of LACTB expression were obtained from Shanghai Outdo Biotech Co., Ltd. (Shanghai, People’s Republic of China; Category no: HPan-Ade170Sur-01), they did not record the corresponding information about R0 and R1. Therefore, we cannot add the above information in Table S2 and Table S4. However, the survival analysis of R0/R1 patients stratified by LACTB mRNA expression were supplied in supplement figure 4.

**Reviewer #2:**

**1.- your Statistical analysis lists a few metrics: OS, DSS, DFI, PFI, NES etc.. these need to be elucidated. Many of them are not expanded until later in the Supplementary. It will help if these terms are explained when they are first introduced in the manuscript to avoid any confusion.**

We have annotated all abbreviations where they first appear

**2. The use of 'Samples' and 'patients' is constantly switched back and forth. It would be preferable if the authors consistently used samples throughout the paper.**

We have changed all the 'Samples' in the original text to 'patients'

**3. Table 1: change Number of sample size to either "Number of samples" or "Sample size" or "percentage of samples"**

The issues mentioned above have been modified

**4. Table 2: Please elucidate further on table2. You show how LACTB expression shows a significant association with Vital status, but it would help if you could further elucidate on what the implications are.. or drive conclusions on what the p-value shows in this table.**

A higher percentage of patients in high LACTB mRNA expression group (61.6%) were decreased compared to the low LACTB mRNA expression group (43.3%) (P = 0.0199). These results suggest that LACTB may be a prognostic factor for PAAD.

**5. it would help strengthen the paper if you could provide a power analysis: that shows the power of your test based on your sample size.**

The power of our test has been supplied in table2
